# Comparative efficacy and safety of bupivacaine versus articaine in third molar surgery: a systematic review and meta-analysis of randomized controlled trials

**DOI:** 10.2340/aos.v85.45787

**Published:** 2026-06-03

**Authors:** Zhimei Zeng, Linlin Wang, Guanfu Ding, Zijun Zeng

**Affiliations:** a Stomatology Department, Affiliated Hospital Group of Guangdong Medical University Panyu He Xian Memorial Hospital, Guangzhou, Guangdong, China; b Neurosurgery Department, Affiliated Hospital Group of Guangdong Medical University Panyu He Xian Memorial Hospital, Guangzhou, Guangdong, China; c Anesthesia Surgery Center, The First Affiliated Hospital of Gannan Medical University, Ganzhou, Jiangxi, China

**Keywords:** bupivacaine, articaine, third molar surgery, local anesthesia, postoperative pain

## Abstract

**Introduction:**

This review systematically compares the efficacy and safety of bupivacaine and articaine in third molar surgery, with a focus on postoperative pain control, duration of analgesia, intraoperative outcomes, and hemodynamic stability.

**Methods:**

PubMed, Scopus, Web of Science, and CENTRAL were searched through December 24^th^, 2024, for randomized controlled trials (RCTs) comparing bupivacaine and articaine in third molar surgery. Primary outcomes included postoperative pain intensity (visual analog scale [VAS]), duration of postoperative analgesia, and need for rescue analgesia. Secondary outcomes included anesthesia onset time, intraoperative bleeding, operative time, and hemodynamic parameters. Meta-analysis was performed using a random-effects model.

**Results:**

A total of 11 RCTs involving 749 patients (bupivacaine: 367, articaine: 382) were included. Bupivacaine significantly reduced postoperative pain scores at multiple time points, with the greatest effect observed at 4 h postoperatively (mean difference [MD] = −2.59; 95% confidence interval [CI]: −3.41, −1.77; *I*^2^ = 0%). The duration of postoperative analgesia was comparable between groups (MD = 91.22 min; 95% CI: −5.13, 187.57; *I*^2^ = 99.68%), with sensitivity analysis confirming this effect. Articaine exhibited a significantly faster onset of anesthesia (MD = 0.74 min; 95% CI: 0.36, 1.12; *I*^2^ = 81.05%), while intraoperative pain scores, surgical difficulty, and operative time were comparable between groups. No significant differences were found in hemodynamic parameters, suggesting similar safety profiles.

**Conclusion:**

Bupivacaine provides superior postoperative pain control and prolonged analgesia, making it advantageous for extended pain relief in third molar surgery. However, articaine’s faster onset may enhance surgical efficiency. Both anesthetics demonstrated comparable intraoperative efficacy and safety.

## Introduction

Third molar extraction is one of the most common surgical procedures in oral and maxillofacial practice, frequently associated with significant postoperative pain, swelling, and discomfort [1]. Effective pain management is crucial to enhancing patient recovery, minimizing reliance on opioid and non-opioid analgesics, and reducing overall morbidity [2]. Local anesthesia remains the cornerstone of perioperative pain control in third molar surgery, with bupivacaine and articaine being two of the most commonly used anesthetics [3].

Bupivacaine, a long-acting amide anesthetic, is widely utilized for its prolonged duration of analgesia, which can reduce postoperative pain intensity and delay the need for rescue analgesia. However, its longer onset time may be a limiting factor in surgical settings requiring rapid anesthesia induction [4]. Conversely, articaine, a thiophene-derived amide anesthetic, has been favored for its rapid onset and superior tissue diffusion, making it particularly advantageous in infiltration techniques [5]. While some studies suggest articaine may provide superior intraoperative pain control [6, 7], concerns have been raised regarding its potential association with neurotoxicity, particularly when used in mandibular nerve blocks [8].

Despite the widespread use of bupivacaine and articaine in third molar surgery, comparative evidence regarding their relative efficacy and safety remains inconsistent. While some trials suggest bupivacaine may provide longer postoperative analgesia, others highlight articaine’s faster onset as a major advantage in clinical practice [9–11]. Moreover, discrepancies in study methodologies, patient populations, and outcome measures have led to variability in reported findings, necessitating a systematic synthesis of the available evidence.

Previous reviews have primarily focused on articaine versus lidocaine [12], while comparisons between bupivacaine and articaine remain limited [13]. Given that these two anesthetics differ not only in pharmacokinetics but also in their intraoperative and postoperative effects, an evidence-based assessment of their comparative performance is essential to guide clinical decision-making in oral surgery.

This systematic review and meta-analysis aimed to compare the efficacy and safety of bupivacaine versus articaine in third molar surgery. Specifically, we sought to evaluate postoperative pain intensity at multiple time points, assess the duration of postoperative analgesia and need for rescue analgesia, compare the onset time of anesthesia and intraoperative pain control, and analyze secondary outcomes, including operative time, intraoperative bleeding, hemodynamic effects, and overall safety.

## Materials and methods

### Study design

This study was conducted as a systematic review and meta-analysis to compare the efficacy and safety of bupivacaine and articaine in third molar surgery. The methodology adhered to the Preferred Reporting Items for Systematic Reviews and Meta-Analyses (PRISMA) guidelines.

### Search strategy and data sources

A comprehensive literature search was performed across four major electronic databases: PubMed, Scopus, Web of Science (WOS), and CENTRAL. Furthermore, Google Scholar (first 200 records to maintain relevance [14]) was searched independently to identify relevant grey literature. The search was conducted using the following query: “Bupivacaine[tiab] AND random[tiab] AND molar[tiab]”. The detailed search query can be found in Table S1. The original search was done on December 24th, 2024.

### Eligibility criteria

Studies were included if they met the following criteria (according to the PICOS framework [15]):

- Population: Patients undergoing third molar surgery
- Intervention: Administration of bupivacaine as a local anesthetic
- Comparator: Administration of articaine as a local anesthetic
- Outcomes: Studies reporting at least one of the predefined outcomes related to efficacy or safety
- Study Design: Randomized controlled trials (RCTs)

The following studies were excluded:

- Non-randomized studies, case reports, editorials, or reviews
- Studies without a direct comparison between bupivacaine and articaine
- Studies lacking extractable quantitative data

### Outcome measures

The primary outcomes included postoperative pain (measured by visual analog scale [VAS], standardized to a 10-point scale), duration of postoperative analgesia (min), the need for rescue analgesia, and the time to first rescue analgesia (h). Meanwhile, secondary outcomes included: onset time of anesthesia (min), the duration of postoperative anesthesia (min), the duration of soft tissue anesthesia (min), operative time (min), surgical difficulty score (1 = easy; 2 = normal; 3 = complicated), intraoperative bleeding score (1 = minimal; 2 = normal; 3 = excessive), and the total amount of rescue analgesia (mg). In addition, the impact of the given anesthetics on hemodynamic parameters (systolic and diastolic blood pressure, heart rate) was investigated.

### Study selection and data extraction

Two independent reviewers screened the titles and abstracts of all retrieved studies. Full-text reviews were conducted for studies meeting the eligibility criteria. Discrepancies were resolved by consensus or a third reviewer. Data extraction was performed using a pre-defined template, collecting information on study characteristics (trial registration, blinding, country and year of investigation), participant demographics (sample size, age, gender), intervention details (type of anesthetic and concentration), and outcome measures (listed above).

### Risk of bias assessment

The risk of bias was evaluated using the Cochrane Risk of Bias 2.0 tool for RCTs, assessing domains including randomization, allocation concealment, blinding, incomplete outcome data, and selective reporting. Studies were categorized as low risk, some concerns, or high risk of bias.

### Certainty of evidence assessment

The certainty of evidence for each outcome was assessed using the Grading of Recommendations Assessment, Development and Evaluation (GRADE) approach. RCTs were initially rated as high certainty and downgraded based on risk of bias, inconsistency, and imprecision.

### Statistical analysis

Meta-analyses were conducted using a random-effects model employing the restricted maximum likelihood method (REML) estimator. Treatment effects were reported as mean differences (MD) or odds ratios (OR) with corresponding 95% confidence intervals (CI). No small-sample or Hartung–Knapp adjustments were applied. A subgroup analysis was done based on follow-up duration (for postoperative pain). Heterogeneity was assessed using *I*² statistics. Sensitivity analyses were conducted for outcomes with *I*² > 50% (and *p* < 0.05), and publication bias assessment was not feasible due to the limited number of included studies (<10 per outcome). Statistical analyses were performed using STATA Software (Version 18, Stata Corp, Texas, USA).

## Results

### Literature search results

**Figure 1:**
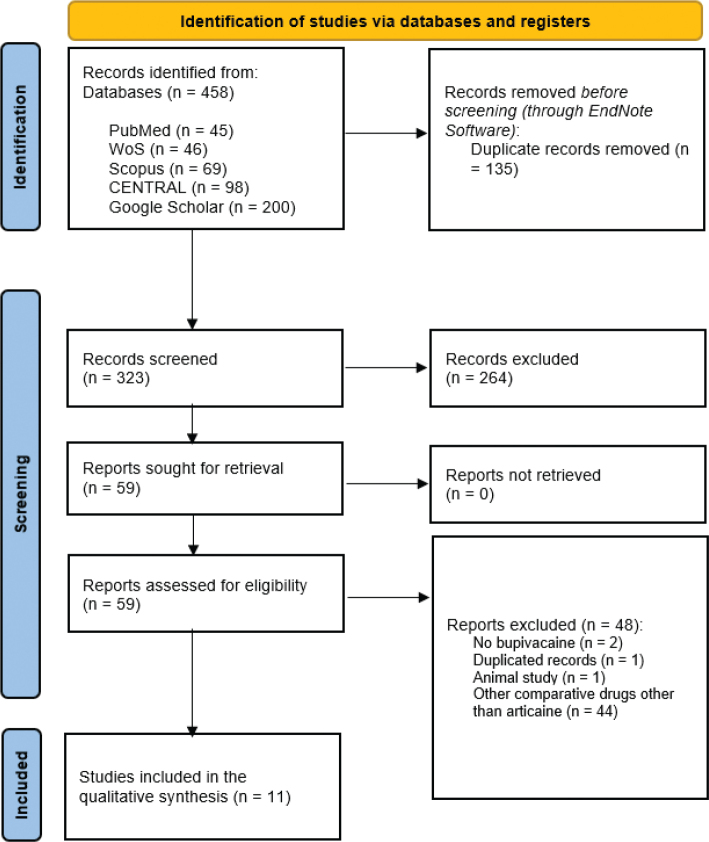
A PRISMA flow diagram showing the results of the database search and screening phases.

The initial database search identified 458 records from PubMed (*n* = 45), WOS (*n* = 46), Scopus (*n* = 69), CENTRAL (*n* = 98), and Google Scholar (*n* = 200). After removing 135 duplicate records, 323 studies remained for title and abstract screening. Following screening, 264 studies were excluded based on irrelevance to the study objectives. A total of 59 full-text articles were assessed for eligibility. Of these, 48 studies were excluded for the following reasons: a lack of bupivacaine as an intervention (*n* = 2), duplicated records (*n* = 1), animal study (*n* = 1), and the use of comparative anesthetics other than articaine (*n* = 44). Finally, 11 RCTs met the inclusion criteria and were included in the meta-analysis [7, 9–11, 16–22]. The study selection process is shown in Figure 1.

### Baseline characteristics of included RCTs

A total of 11 RCTs comparing bupivacaine and articaine in third molar surgery were included in this systematic review. All studies were double-blinded, except for two trials that used a triple-blind design. None of the studies were prospectively registered, except for Olmedo-Gaya [10] (ACTRN12617001138370) and Tokuc [7] (NCT04645888). The included studies were conducted across multiple countries, including the USA (*n* = 1), India (*n* = 3), Brazil (*n* = 1), Spain (*n* = 4), Iraq (*n* = 1), and Turkey (*n* = 1).

The sample sizes varied across studies, ranging from 18 to 50 patients per group, with a total of 749 patients analyzed (367 in the bupivacaine group and 382 in the articaine group). The mean or median age of participants was reported with interquartile ranges in some studies, with most participants being young adults. The proportion of male participants ranged from 24% to 60%, reflecting a mixed-gender distribution. The full demographic and methodological details of the included studies are summarized in Table 1.

**Table 1:** Baseline demographic characteristics of included randomized trials comparing bupivacaine to articaine in third molar surgery anesthesia.

Author (YOP)	Design	Blinding	Registration	Country	YOI	Group	Sample Size	Age; mean (SD)	Male
Abdulwahab (2009) [16]	RCT	Double blinding	Not registered	USA	–	Bupivacaine 0.50%	18	24.9 (18–53)*	6 (33.33%)
						Articaine (A100) 4%	18		6 (33.33%)
						Articaine (A200) 4%	18		6 (33.33%)
Aggarwal (2017) [9]	RCT	Double blinding	Not registered	India	–	Bupivacaine 0.50%	34	34 (27–41)#	19 (55.88%)
						Articaine 4%	31	38 (29–45)#	16 (42.11%)
Ahmed (2021) [20]	RCT	Double blinding	Not registered	India	–	Bupivacaine 0.50%	25	22.17 (6.10)	13 (52%)
						Articaine 4%	25	21.06 (5.69)	13 (52%)
Gregorio (2008) [17]	RCT	Double blinding	Not registered	Brazil	–	Bupivacaine 0.50%	50	21.84 (0.65)	21 (42%)
						Articaine (A200) 4%	50		21 (42%)
Kaur (2024) [18]	RCT	Double blinding	Not registered	India	–	Bupivacaine 0.50%	25	29.52 (18–50)*	–
						Articaine 4%	25		–
Olmedo-Gaya (2018) [10]	RCT	Double blinding	ACTRN12617001138370	Spain	April 2014 – March 2016	Bupivacaine 0.50%	50	22.17 (6.10)	12 (24%)
						Articaine 4%	50	21.06 (5.69)	14 (28%)
Pellicer-Chover (2013) [19]	RCT	Double blinding	Not registered	Spain	November 2009 – May 2010	Bupivacaine 0.50%	36	23.1 (6)*	12 (33.33%)
						Articaine 4%	36		12 (33.33%)
Sancho-Puchades (2012) [11]	RCT	Triple blinding	Not registered	Spain	–	Bupivacaine 0.50%	18	23.8 (5.0)*	7 (38.89%)
						Articaine 4%	18		7 (38.89%)
Tenglikar (2022) [21]	RCT	Double blinding	Not registered	Iraq	June 2017 – October 2019	Bupivacaine 0.50%	50	(20–50)*	30 (60%)
						Articaine 4%	50		27 (54%)
Tokuc (2021) [7]	RCT	Triple blinding	NCT04645888	Turkey	–	Bupivacaine 0.50%	26	22.1 (4.0)	6 (23.08%)
						Articaine 4%	26		6 (23.08%)
Trullenque-Eriksson (2011) [22]	RCT	Double blinding	Not registered	Spain	October 2008 – March 2009	Bupivacaine 0.50%	35	24.47	11 (31.43%)
						Articaine 4%	35		11 (31.43%)

RCT: randomized controlled trial; YOP: year of publication; YOI: year of investigation; SD: standard deviation; USA: United States of America.

# data are reported as median (interquartile range);

* data are reported as median (range).

### Risk of bias of included RCTs

**Figure 2:**
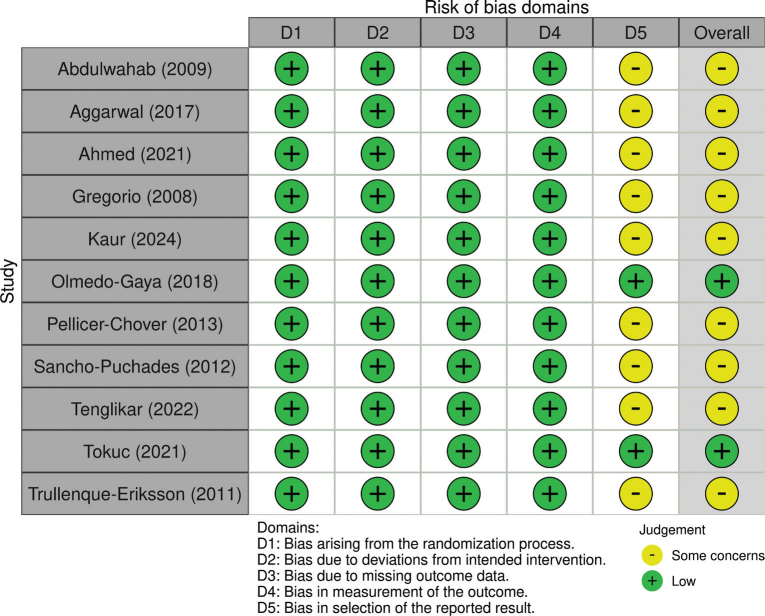
A summary of the risk of bias of included RCTs using the revised Cochrane risk of bias tool.

All but two trials had some concerns (Figure 2). The remaining two RCTs had low risk of bias. The main concern was with the lack of a registered protocol to ascertain reporting validity.

### GRADE certainty

Given that most trials had some concerns regarding risk of bias and several outcomes demonstrated substantial heterogeneity and wide CIs, the certainty of evidence ranged from moderate to very low (Table 2).

**Table 2:** A summary of findings and certainty of evidence (GRADE) for the comparison between bupivacaine and articaine in third molar surgery.

Outcome	RCTs	Effect measure (95% CI)	*I* ^2^	Key issues	GRADE certainty
Pain score – 4 h	2	MD = −2.59 (−3.41, −1.77)	0%	RoB (–1); small number of trials (imprecision –1)	Low
Pain score – 24 h	8	MD = −0.46 (−0.86, −0.05)	56.19%	RoB (–1); inconsistency (–1)	Low
Need for rescue analgesia	3	OR = 1.24 (0.63, 2.10)	0%	RoB (–1); imprecision (CI crosses null, –1)	Low
Time to first rescue analgesia	2	MD = 5.13 (−15.58, 25.85)	63.79%	RoB (–1); inconsistency (–1); imprecision (–1)	Very Low
Duration of postoperative analgesia	5	MD = 91.22 (−5.13, 187.57)	99.68%	RoB (–1); very serious inconsistency (–2); imprecision (–1)	Very Low
Sensitivity analysis	4	MD = 32.67 (3.36, 61.98)	↓ (post-exclusion)	RoB (–1); residual imprecision (–1)	Low
Duration of postoperative anesthesia	3	MD = 183.03 (47.42, 318.65)	98.91%	RoB (–1); very serious inconsistency (–2)	Very Low
Duration of soft tissue anesthesia	4	MD = 137.20 (47.22, 227.18)	97.62%	RoB (–1); very serious inconsistency (–2)	Very Low
Onset time of anesthesia	5	MD = 0.74 (0.36, 1.12)	81.05%	RoB (–1); serious inconsistency (–2)	Very Low
Intraoperative bleeding	3	MD = 0.05 (−0.01, 0.11)	0%	RoB (–1); imprecision (–1)	Low
Operative time	4	MD = 2.10 (1.52, 2.69)	0%	RoB (–1); imprecision (–1)	Low
Surgical difficulty score	3	MD = 0.02 (−0.04, 0.09)	0%	RoB (–1); imprecision (–1)	Low
SBP	5	MD = 0.58 (−2.75, 3.90)	34.98%	RoB (–1); imprecision (–1)	Low
DBP	4	MD = 2.31 (0.19, 4.43)	34.19%	RoB (–1); imprecision (–1)	Low
HR	5	MD = 1.19 (−1.70, 4.07)	0%	RoB (–1); imprecision (–1)	Low

RCT: randomized controlled trial; CI: confidence interval; MD: mean difference; OR: odds ratio; I²: I-squared statistic for heterogeneity; RoB: risk of bias; GRADE: Grading of Recommendations Assessment, Development and Evaluation; SBP: systolic blood pressure; DBP: diastolic blood pressure; HR: heart rate.

### Postoperative pain

**Figure 3:**
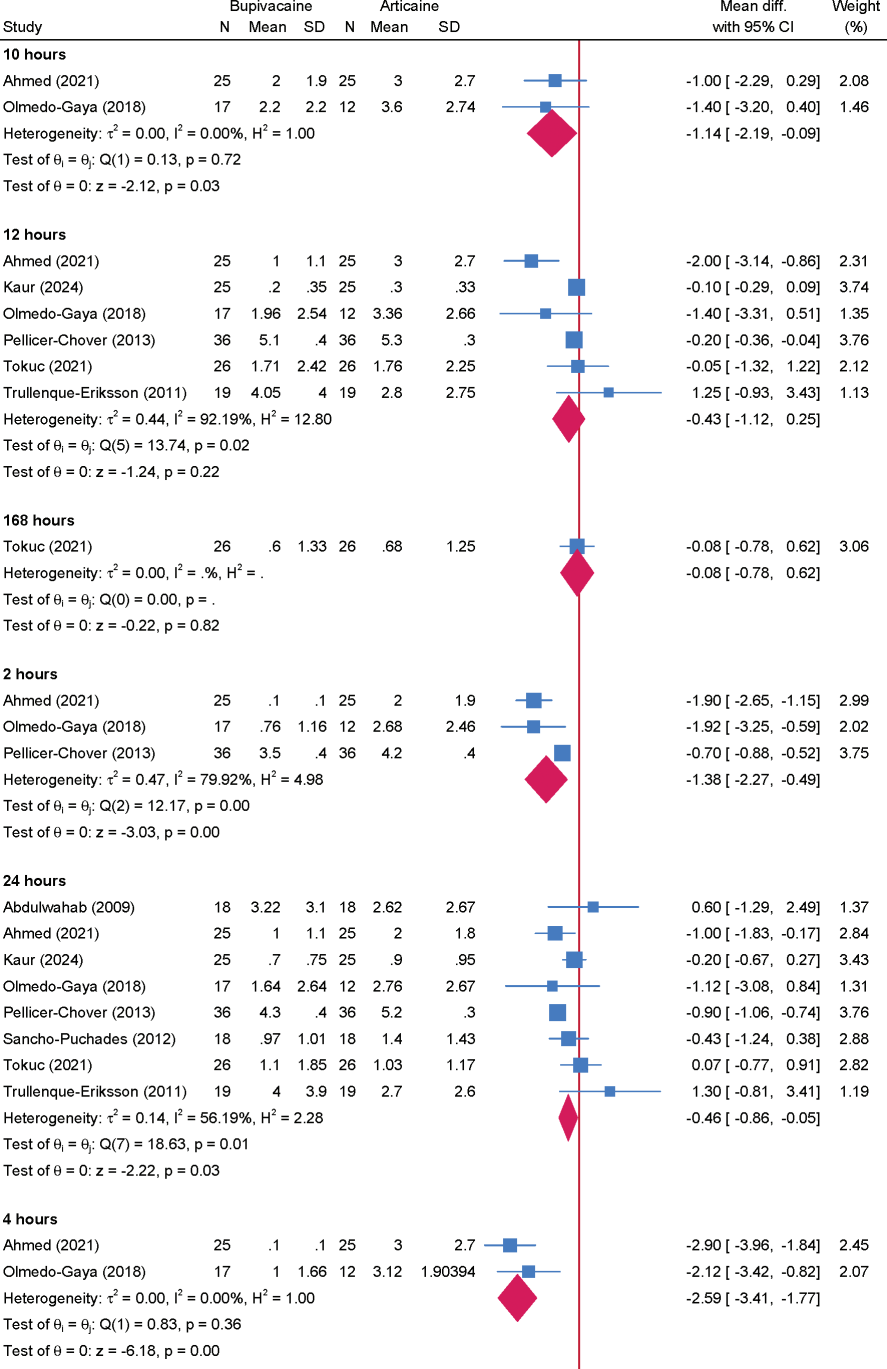

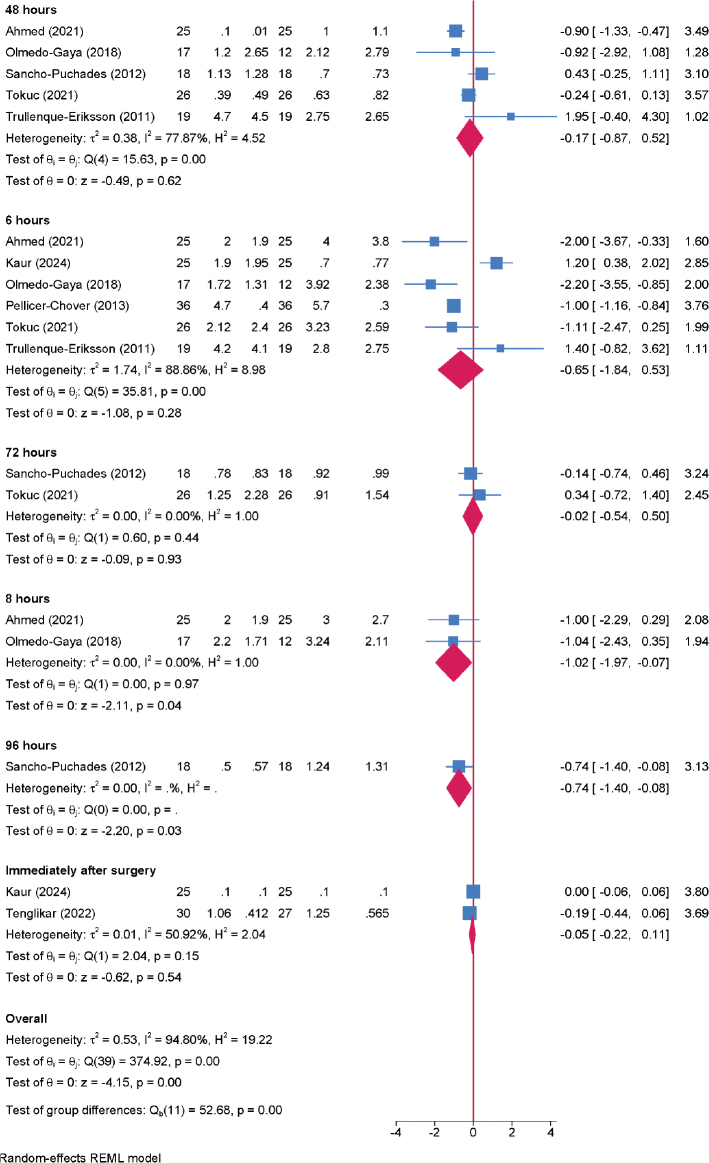
Forest plot showing the difference in postoperative pain score between bupivacaine and articaine stratified by the assessment timepoint.

Pain scores were assessed at multiple time points following surgery. A total of nine RCTs involving 420 patients contributed to the analysis (Figure 3). Bupivacaine showed significant reduction in pain scores compared to articaine at 2, 4, 8, 10, and 24 h. The greatest reduction was observed at 4 h (2 RCTs; MD = −2.59; 95% CI: −3.41; −1.77; *I*^2^ = 0%; low certainty) with a progressive decline over time reaching the lowest reduction at 24 h (9 RCTs; MD = −0.46; 95% CI: −0.86; −0.05; *I*^2^ = 56.19%; low certainty). No significant differences were observed at longer intervals (48 to 168 h).

### Need for rescue analgesia and time to first rescue analgesia

Three RCTs with 224 patients compared the need for rescue analgesia between bupivacaine and articaine (Figure S1). The analysis showed no significant difference (OR = 1.24; 95% CI: 0.63, 2.10; *I*² = 0.00%; low certainty), suggesting similar analgesic efficacy between the two anesthetics.

Two RCTs with 108 patients evaluated the time to first rescue analgesia use (Figure S2). While bupivacaine showed a longer time to first analgesic requirement, the difference was not statistically significant (2 RCTs, 54 patients; MD = 5.13 h; 95% CI: −15.58, 25.85; *I*² = 63.79%; very low certainty). Sensitivity analysis was infeasible due to small sample.

### Duration of postoperative analgesia and anesthesia

**Figure 4:**
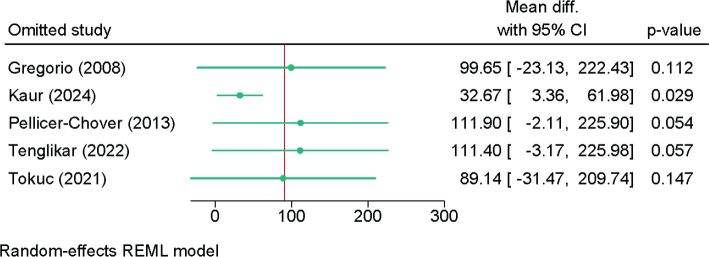
Sensitivity analysis of the difference in the duration of postoperative analgesia between bupivacaine and articaine.

Five RCTs with 287 patients assessed the duration of postoperative analgesia (Figure S3). Bupivacaine showed comparable duration of analgesia compared to articaine (MD = 91.22 min; 95% CI: −5.13, 187.57; *I*² = 99.68%; very low certainty). However, due to substantial heterogeneity, sensitivity analyses were conducted. The Galbraith plot showed that the study of Kaur et al. [18] was an outlier (Figure S4). However, the sensitivity analysis showed a significant increase in the duration of postoperative analgesia in bupivacaine compared to articaine after excluding Kaur et al. (4 RCTs; MD = 32.67; 95% CI: 3.36–61.98; low certainty) (Figure 4).

**Figure 5:**
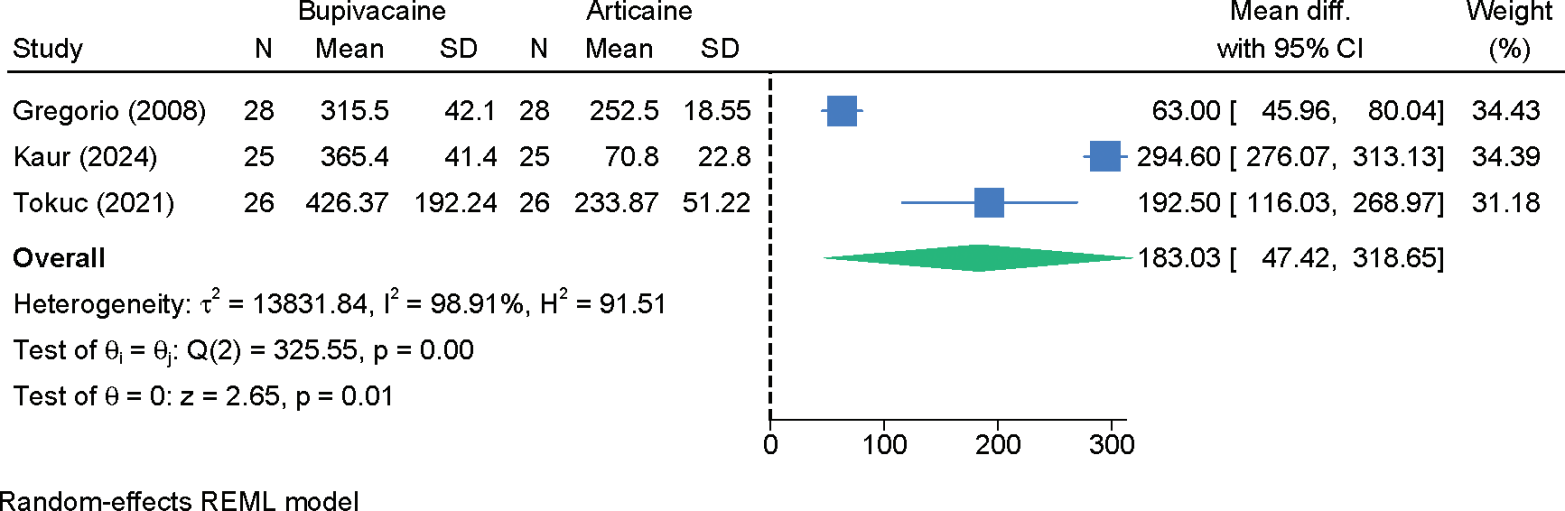
Forest plot showing the difference in the duration of postoperative anesthesia between Bupivacaine and articaine.

Three RCTs with 158 patients assessed the duration of postoperative anesthesia. Bupivacaine provided significantly longer anesthesia compared to articaine (MD = 183.03 min; 95% CI: 47.42, 318.65; *I*² = 98.91%; very low certainty) (Figure 5). Sensitivity analyses confirmed the direction of the effect, though with some variation in magnitude following the exclusion of the study of Gregorio et al. [17] (2 RCTs; MD = 250.57; 95% CI: 151.46 – 349.67) (Figure S5).

### Duration of soft tissue anesthesia

**Figure 6:**
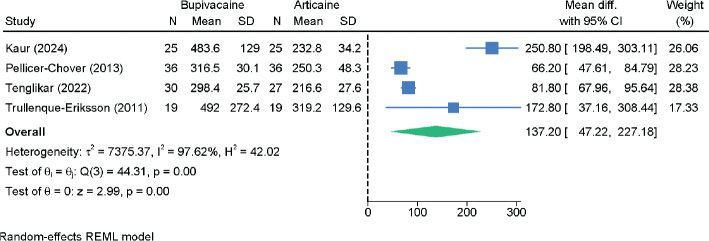
Forest plot showing the difference in the duration of soft tissue anesthesia between bupivacaine and articaine.

Four RCTs with 220 patients assessed the duration of soft tissue anesthesia (Figure 6). Bupivacaine significantly prolonged soft tissue anesthesia compared to articaine (MD = 137.20 min; 95% CI: 47.22, 227.18; *I*² = 97.62%; very low certainty). Sensitivity analysis results remained consistent but showed some variation in the estimated effect size (Figure S6).

### Onset time of anesthesia

**Figure 7:**
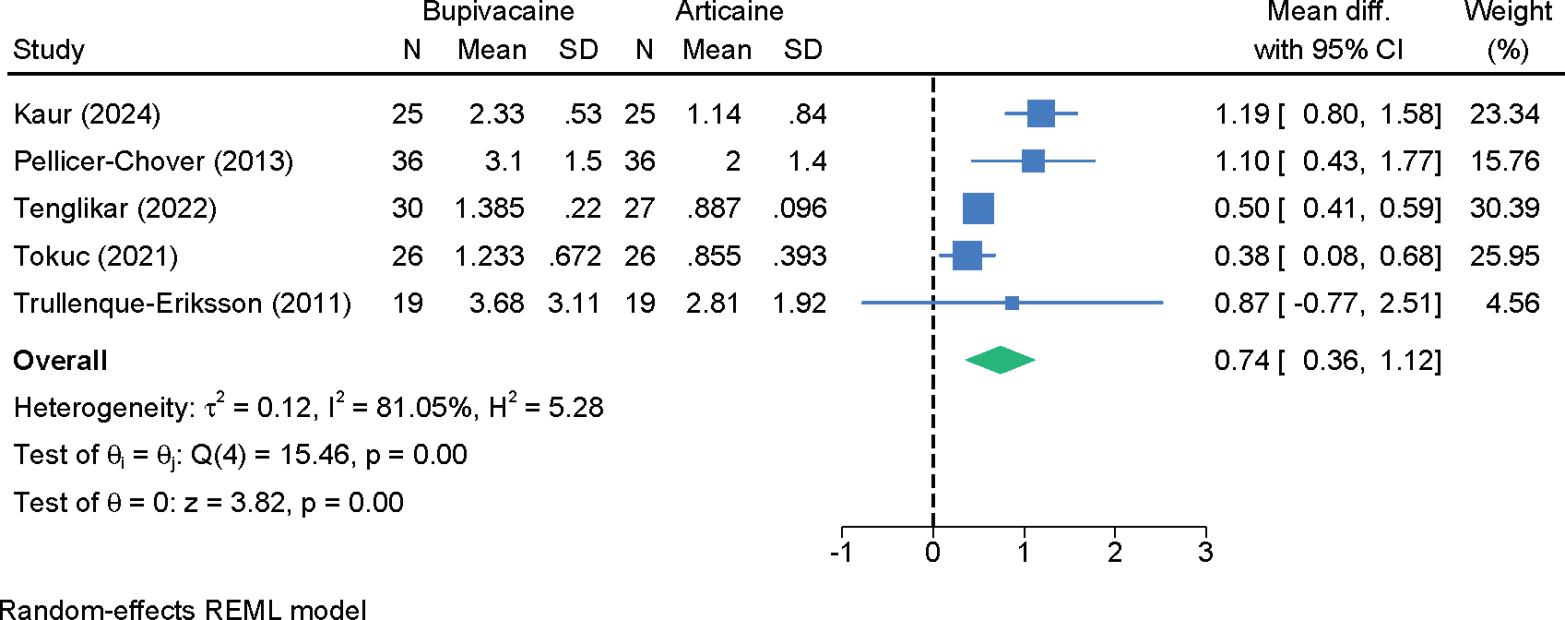
Forest plot showing the difference in the onset time of anesthesia between bupivacaine and articaine.

Five RCTs with 269 patients evaluated the onset time of anesthesia (Figure 7). Bupivacaine had a significantly slower onset compared to articaine (MD = 0.74 min; 95% CI: 0.36 – 1.12; *I*² = 81.05%; very low certainty), confirming that articaine induces anesthesia more rapidly. Sensitivity analyses maintained statistical significance across models (Figure S7).

### Intraoperative bleeding, operative time, and surgical difficulty score

Three RCTs with 158 patients compared intraoperative bleeding scores between the two anesthetics (Figure S8). No significant difference was observed (MD = 0.05; 95% CI: −0.01, 0.11; *I*² = 0.00%; low certainty), indicating comparable hemostatic effects.

**Figure 8:**
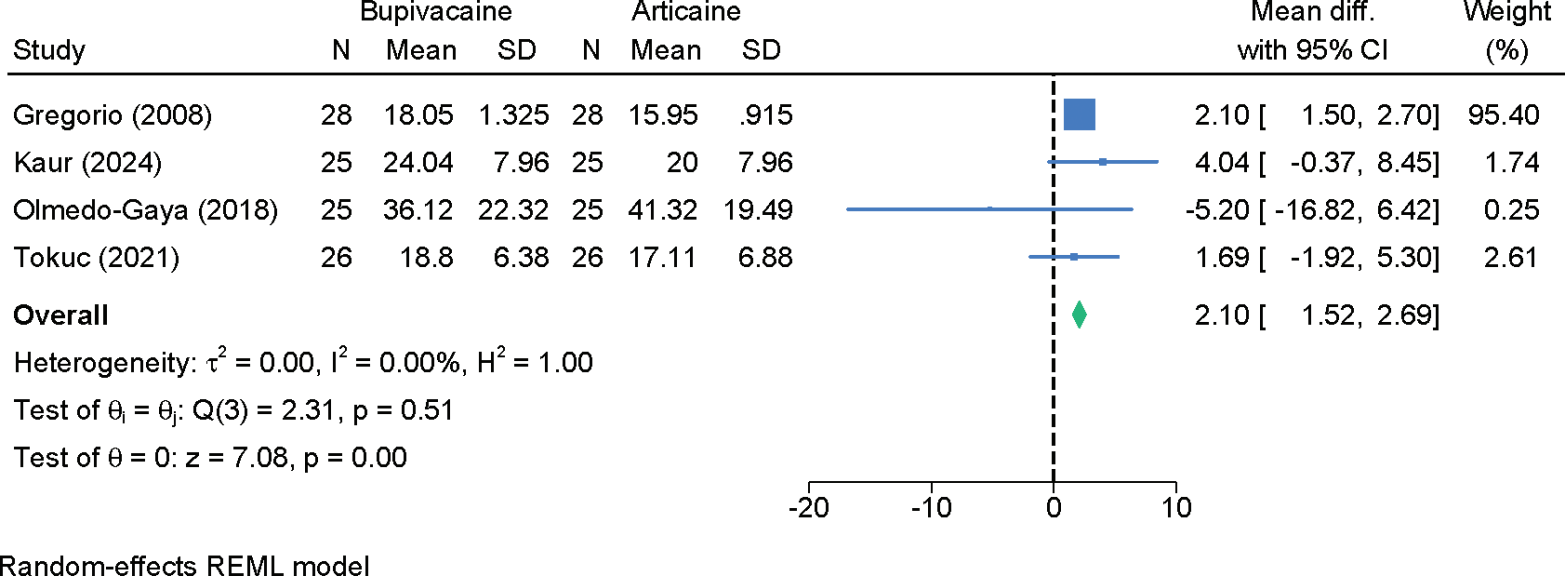
Forest plot showing the difference in operative time between bupivacaine and articaine.

Four RCTs with 208 patients analyzed operative time (Figure 8). Bupivacaine was associated with a slightly longer operative duration, though the difference was not clinically relevant (MD = 2.10 min; 95% CI: 1.52, 2.69; *I*² = 0.00%; low certainty).

Three RCTs with 158 patients assessed the surgical difficulty score and found no significant difference between groups (MD = 0.02; 95% CI: −0.04, 0.09; *I*² = 0.00%; low certainty) (Figure S9).

### Systolic blood pressure, diastolic blood pressure, and heart rate

Five RCTs with 274 patients evaluated the effects on systolic blood pressure (Figure S10). No significant difference was found (MD = 0.58 mmHg; 95% CI: −2.75, 3.90; *I*² = 34.98%; low certainty), indicating similar hemodynamic profiles between anesthetics.

**Figure 9:**
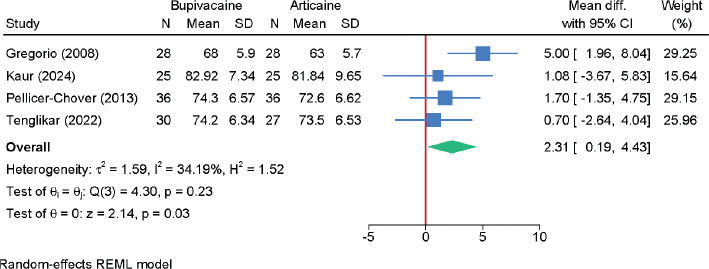
Forest plot showing the difference in postoperative diastolic blood pressure between bupivacaine and articaine.

Four RCTs with 238 patients assessed diastolic blood pressure (Figure 9). Bupivacaine was associated with a small but statistically significant increase (MD = 2.31 mmHg; 95% CI: 0.19, 4.43; *I*² = 34.19%; low certainty), though the clinical relevance of this effect is debatable.

Five RCTs with 274 patients compared heart rate changes (Figure S11). No significant difference was detected (5 RCTs, 137 patients; MD = 1.19 bpm; 95% CI: −1.70, 4.07; *I*² = 0.00%; low certainty), suggesting that neither anesthetic had a notable effect on heart rate.

## Discussion

### Summary of findings

This systematic review and meta-analysis compared the efficacy and safety of bupivacaine and articaine in third molar surgery. Our findings demonstrate that bupivacaine provides superior postoperative analgesia with comparable duration of effect compared to articaine, reducing early postoperative pain intensity and delaying the need for rescue analgesia. However, articaine showed a faster onset of anesthesia, making it an attractive option for surgical efficiency. The two anesthetics exhibited comparable intraoperative pain control, hemodynamic stability, and overall safety. These results contribute to the ongoing debate regarding the optimal anesthetic choice for third molar extraction and align with previous literature while offering novel insights through meta-analytical synthesis.

### Postoperative pain and analgesia duration

Pain management remains a critical consideration in third molar surgery due to its direct impact on patient recovery and satisfaction. Our analysis revealed that bupivacaine resulted in significantly lower postoperative pain scores at multiple time points, with the greatest reduction at 4 h postoperatively. This aligns with previous studies indicating that bupivacaine’s prolonged action provides extended pain relief [10, 23]. Meanwhile, the duration of analgesia was comparable between both groups; however, given the substantial heterogeneity observed in the analysis, this finding should not be perceived as conclusive [3].

No statistically significant difference was observed between bupivacaine and articaine in the need for rescue analgesia, indicating comparable postoperative analgesic requirements between the two agents. While some trials suggest that articaine may provide slightly better intraoperative pain control, its postoperative effects appear to diminish more rapidly [24]. These findings support the judicious selection of bupivacaine in cases where prolonged analgesia is a priority, particularly for patients with low pain tolerance or limited access to postoperative pain management.

### Onset of anesthesia and intraoperative considerations

A significant limitation of bupivacaine is its slower onset of action, which was confirmed in our meta-analysis. Articaine demonstrated a significantly faster onset, which is in agreement with previous studies comparing local anesthetics in oral surgery [12]. This characteristic makes articaine a preferred choice in procedures requiring rapid anesthetic action, such as high-throughput surgical settings.

Despite this, intraoperative pain scores did not differ significantly between the two anesthetics, reinforcing that both provide adequate surgical anesthesia. Notably, the use of articaine in mandibular blocks has been associated with a higher risk of nerve injury, though this was not a concern in our dataset [25]. Given these factors, a combination strategy – using articaine for its rapid onset followed by bupivacaine for prolonged postoperative analgesia – may be optimal for certain patients [26].

### Hemodynamic and safety considerations

Hemodynamic stability is a key concern in local anesthetic selection, particularly for patients with cardiovascular comorbidities. Our results showed no significant differences in systolic blood pressure, diastolic blood pressure, or heart rate between bupivacaine and articaine. These findings contrast with some previous studies that suggested bupivacaine may have a greater tendency to cause bradycardia due to its prolonged sodium channel blockade [13].

A major safety concern with articaine has been its potential association with increased neurotoxicity when used in mandibular nerve blocks, leading to hypoesthesia or paresthesia [27]. While our study did not assess adverse effects directly, clinicians should be aware of articaine’s potential risks in certain anatomical regions. Nevertheless, recent systematic reviews suggest that the overall risk remains low and does not warrant absolute avoidance [3].

With respect to safety, this analysis was limited to hemodynamic parameters, which did not differ meaningfully between bupivacaine and articaine. Importantly, none of the included trials reported extractable data on adverse events such as paresthesia, prolonged anesthesia, or nerve injury. Therefore, no conclusions can be drawn regarding comparative risks for these outcomes. This represents an important evidence gap and underscores the need for future trials to systematically report clinically relevant adverse events.

### Study’s limitations

This meta-analysis has several limitations. Several outcomes, particularly the duration of postoperative analgesia, demonstrated substantial between-study heterogeneity. Potential clinical and methodological sources of this heterogeneity include variations in surgical technique and difficulty, differences in anesthetic administration protocols (including volume and use of vasoconstrictors), and inconsistencies in the definition and measurement of postoperative analgesia across trials. In addition, perioperative adjunctive medications were variably reported, and in some studies not reported at all, which may have contributed to residual variability in analgesic outcomes. Methodological factors, such as small sample sizes, a lack of prospective trial registration, and differences in follow-up schedules, may have further amplified heterogeneity. These considerations likely explain the high *I*² values observed and underscore the need for cautious interpretation of pooled estimates.

The small number of included RCTs (*n* = 11) restricted subgroup analyses and prevented a reliable publication bias assessment. Although anesthetic concentrations and epinephrine ratios varied across trials (Table S2), the low heterogeneity and limited number of studies precluded meaningful subgroup or meta-regression analyses to assess their modifying effects. An important limitation of this review is the inconsistent and incomplete reporting of perioperative adjunctive medications, such as corticosteroids, NSAIDs, and sedatives. This precluded harmonization of adjunctive regimens across trials and prevented sensitivity analyses excluding studies with potential imbalances. Consequently, residual confounding related to adjunctive analgesia cannot be excluded.

Moreover, variability in outcome reporting posed challenges in data synthesis, with differences in pain measurement scales, rescue analgesia definitions, and intraoperative bleeding scores. The study population primarily consisted of young adults, limiting the generalizability to elderly or medically complex patients. Furthermore, long-term safety outcomes, such as neurotoxicity or prolonged numbness, were not assessed, as most studies focused on immediate postoperative effects.

### Clinical implications and future directions

Beyond statistical significance, the clinical relevance of the observed effect sizes warrants cautious consideration. The reduction in early postoperative pain scores, particularly at 4 h, represents a magnitude that may be perceptible to patients in the immediate postoperative period. However, the attenuation of this effect over time and the low certainty of evidence limit its clinical interpretability. Meanwhile, bupivacaine was associated with comparable analgesia duration to that of articaine, with the wide CIs and substantial heterogeneity observed across studies indicating considerable uncertainty regarding the consistency and magnitude of this effect in routine clinical practice. As such, these findings should be viewed as suggestive rather than definitive and may be most relevant when individualized to patient preferences and procedural context.

Future research should focus on patient-centered outcomes (such as postoperative quality of life and patient-reported satisfaction), combination strategies (exploring the sequential use of articaine (for rapid onset) and bupivacaine (for prolonged action)), and comparative studies (including adjunctive agents, such as dexamethasone or clonidine, which may enhance analgesic efficacy [28]).

## Conclusion

This systematic review and meta-analysis suggest that bupivacaine is associated with lower early postoperative pain scores and longer durations of anesthesia compared with articaine, while articaine demonstrates a faster onset of anesthesia. Intraoperative outcomes and hemodynamic parameters were generally comparable between the two agents. However, these findings should be interpreted with caution due to the small sample sizes of the included trials, substantial heterogeneity across several outcomes, and predominantly low to very low certainty of evidence. Further well-designed, adequately powered randomized trials with standardized outcome reporting are required to clarify the comparative efficacy and safety of bupivacaine and articaine in third molar surgery.

## Supplementary Material





## Data Availability

All data generated or analyzed during this study are included in this article and its supplementary information files.
