# Supplementary material for: Comparative efficacy and safety of bupivacaine versus articaine in third molar surgery: a systematic review and meta-analysis of randomized controlled trials
Source: Acta Odontol Scand. 2026 Jun 3;85:45787. doi: 10.2340/aos.v85.45787 (PMC13241955; doi:10.2340/aos.v85.45787)
Supplement: Supplementary file 1 [file AOS-85-45787-s1.pdf]

Supplementary material has been published as submitted. It has not been copyedited or typeset by Acta Odontologica Scandinavica.

Table S1. The detailed search query used in the literature search

Database	Query	Results
PubMed	(Bupivacaine[tiab]) AND (random*[tiab]) AND (molar[tiab])	45
Scopus	TITLE-ABS-KEY (bupivacaine) AND TITLE-ABS-KEY (random*) AND TITLE-ABS-KEY(molar)	69
Web of Science	TS=(bupivacaine AND random* AND molar)	46
CENTRAL	(bupivacaine):ti,ab AND (random*):ti,ab AND (molar):ti,ab	98
Google Scholar	bupivacaine random molar	200
Total	-	458
After duplicate removal	-	323

Table S2. Reported concentrations of bupivacaine and articaine, and epinephrine ratios, across included randomized controlled trials.

Author (YOP)	Articaine	Bupivacaine
**Abdulwahab (2009) [16]**	4% articaine with 1:200,000 epinephrine	0.5% bupivacaine with 1:200,000 epinephrine
**Aggarwal (2017) [9]**	4% articaine with 1:100,000 epinephrine	0.5% bupivacaine with 1:200,000 epinephrine
**Ahmed (2021) [20]**	4% articaine with 1:100,000 epinephrine	0.5% bupivacaine with 1:200,000 epinephrine
**Gregorio (2008) [17]**	articaine 4 % with 1:200,000 epinephrine	0.5% bupivacaine with 1:200,000 epinephrine
**Kaur (2024) [18]**	4% articaine with 1:200,000 epinephrine	0.5% bupivacaine with 1:200,000 epinephrine
**Olmedo-Gaya (2018) [10]**	4% articaine with 1:100,000 epinephrine	0.5% bupivacaine with 1:200,000 epinephrine
**Pellicer-Chover (2013) [19]**	articaine at 4% (epinephrine 1:100,000	bupivacaine at 0.5% (epinephrine 1:200,000)
**Sancho-Puchades (2012) [11]**	articaine 4 % with 1:200,000 epinephrine	0.5% bupivacaine with 1:200,000 epinephrine
**Tenglikar (2022) [21]**	4% articaine with 1:100,000 epinephrine	0.5% bupivacaine with 1:200,000 epinephrine

**Tokuc (2021) [7]**	4% articaine with 1:200,000 epinephrine	0.5% bupivacaine without epinephrine
**Trullenque-Eriksson (2011) [22]**	articaine 4 % with 1:200,000 epinephrine	0.5% bupivacaine with 1:200,000 epinephrine

YOP: year of publication.
