# Supplementary material for: Comparative efficacy and safety of bupivacaine versus articaine in third molar surgery: a systematic review and meta-analysis of randomized controlled trials
Source: Acta Odontol Scand. 2026 Jun 3;85:45787. doi: 10.2340/aos.v85.45787 (PMC13241955; doi:10.2340/aos.v85.45787)

Supplementary material has been published as submitted. It has not been copyedited or typeset by Acta Odontologica Scandinavica.

Figure 1

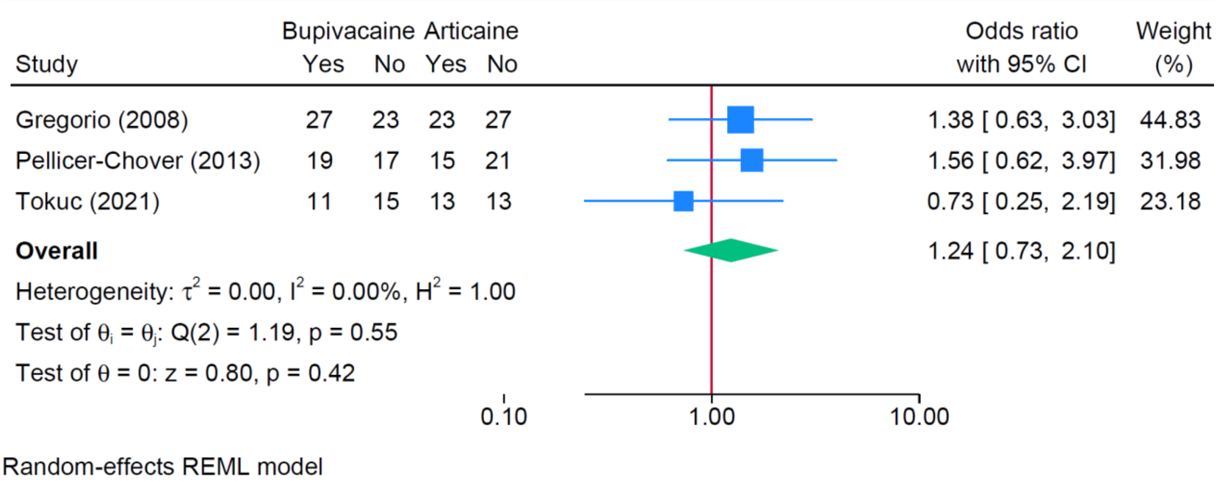

Figure 2

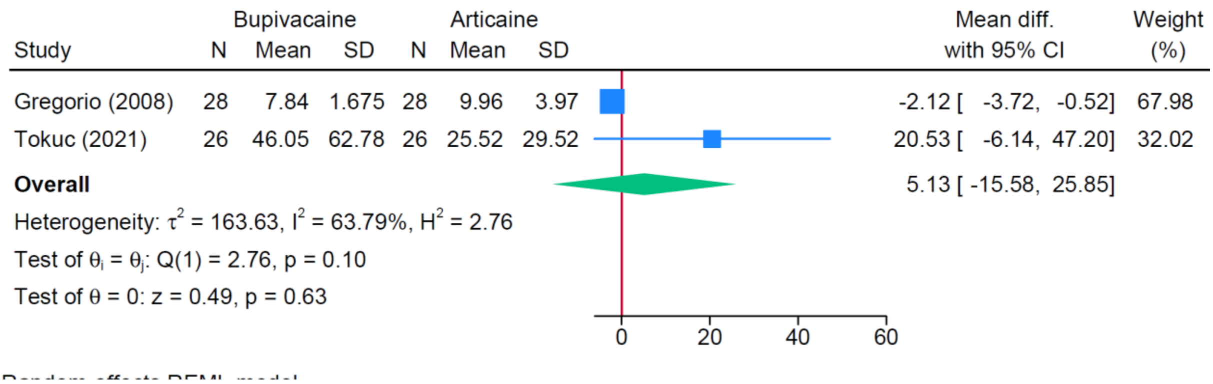

Figure 3

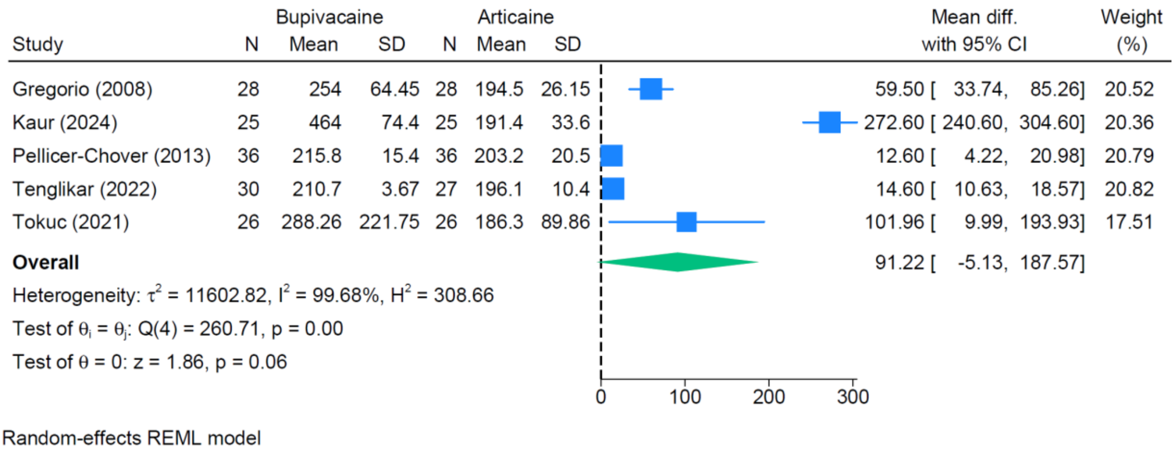

Figure 4

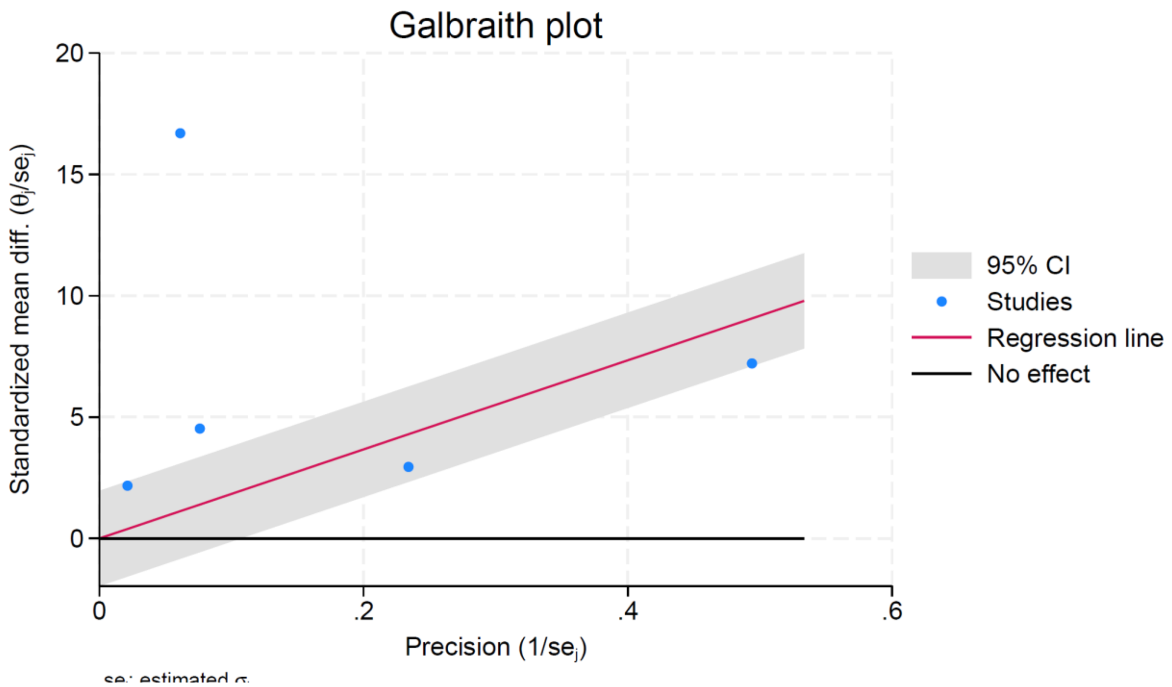

Figure 5

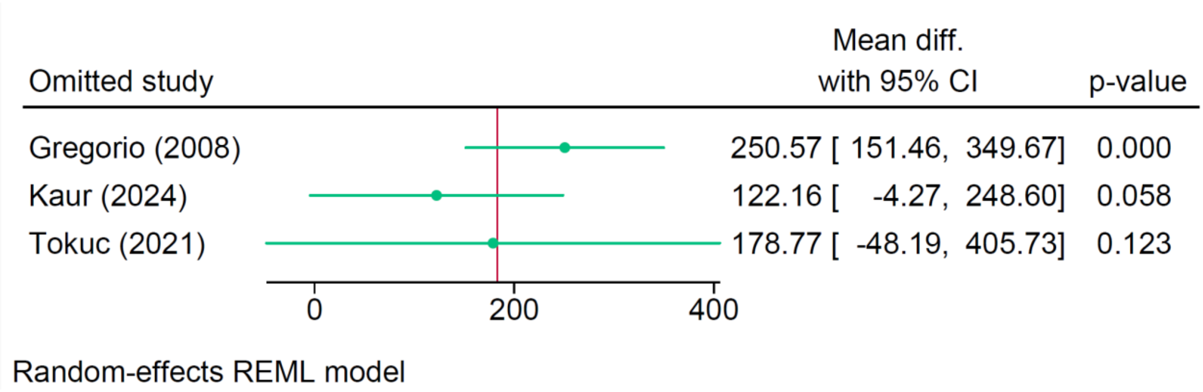

Figure 6

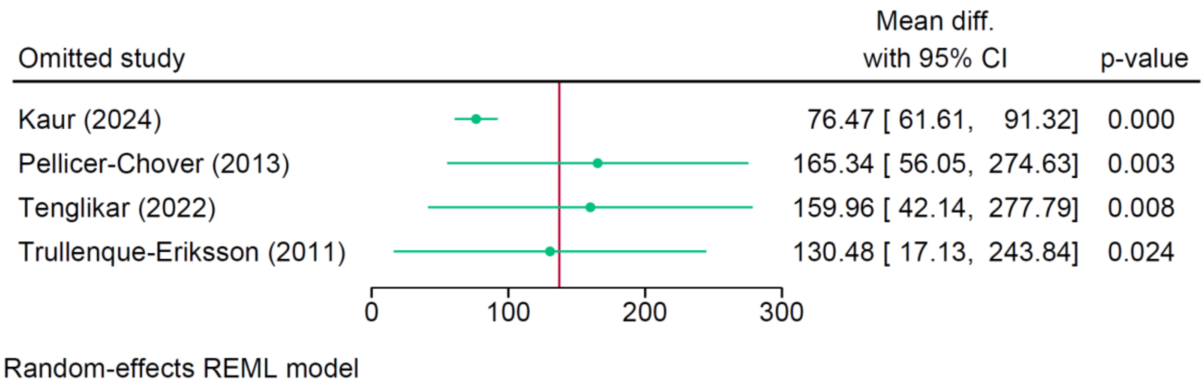

Figure 7

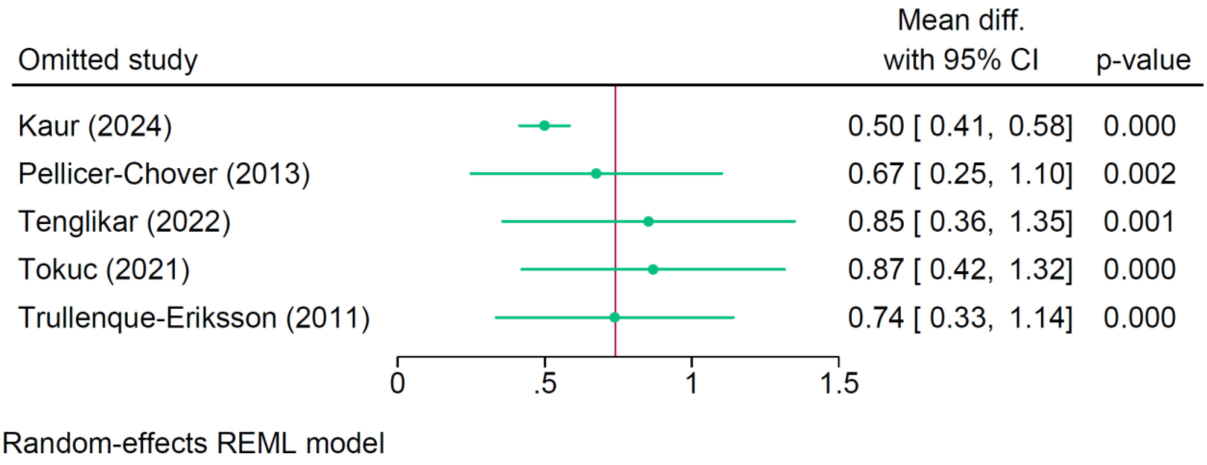

Figure 8

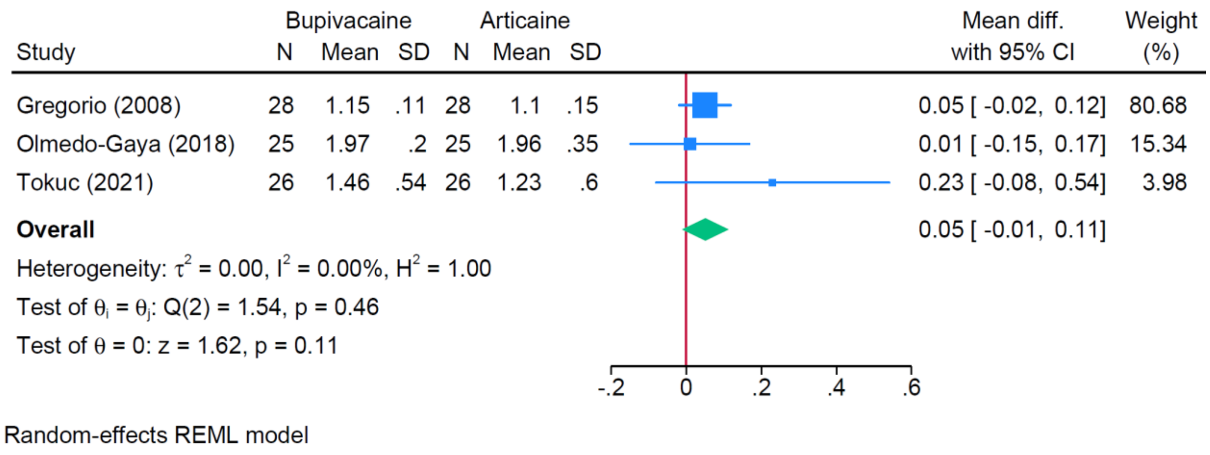

Figure 9

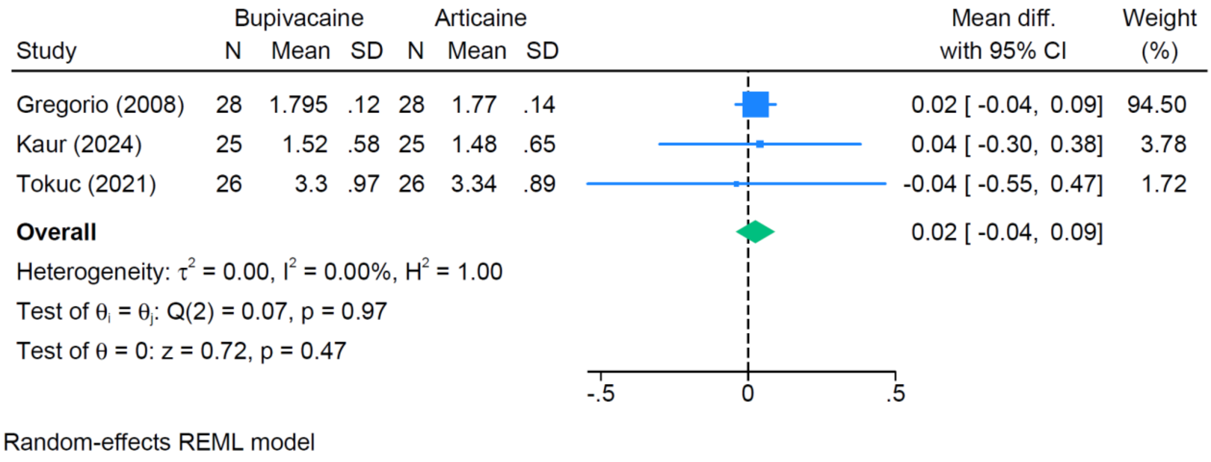

Figure 10

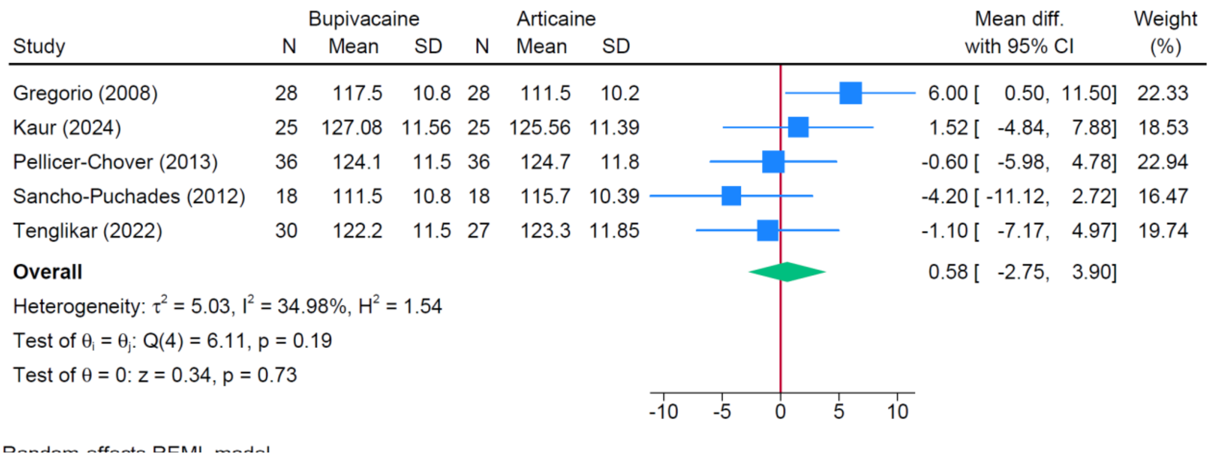

Figure 11

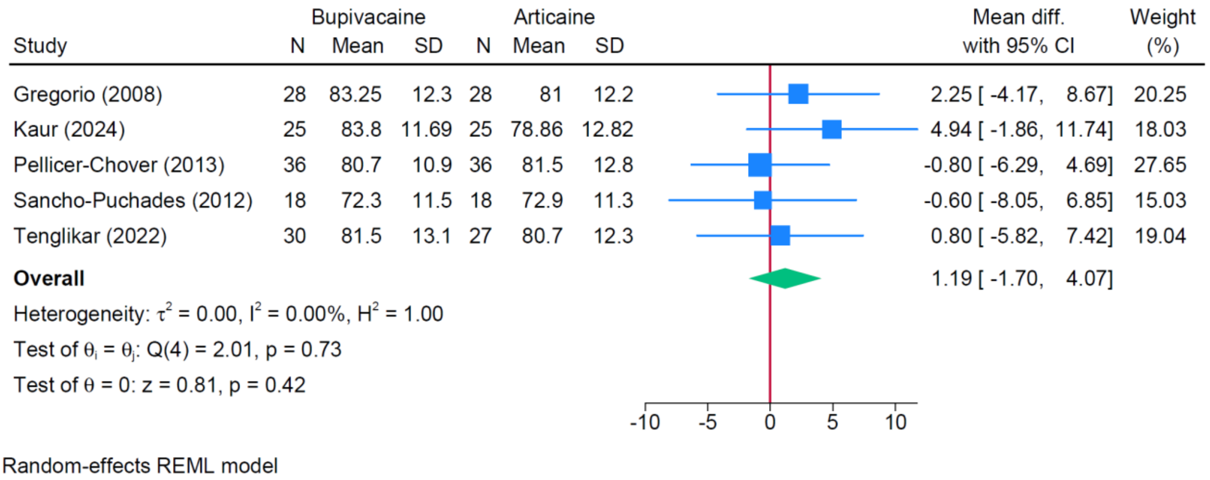

Supplement: Supplementary file 2 [file AOS-85-45787-s2.pdf]
